# Response of Skin-Derived and Metastatic Human Malignant Melanoma Cell Lines to Thymoquinone and Thymoquinone-Loaded Liposomes

**DOI:** 10.3390/pharmaceutics14112309

**Published:** 2022-10-27

**Authors:** Patrycja Kłos, Magdalena Perużyńska, Magdalena Baśkiewicz-Hałasa, Paulina Skupin-Mrugalska, Małgorzata Majcher, Magdalena Sawczuk, Bartosz Szostak, Marek Droździk, Bogusław Machaliński, Dariusz Chlubek

**Affiliations:** 1 Department of Biochemistry and Medical Chemistry, Pomeranian Medical University in Szczecin, Powstańców Wlkp. 72, 70-111 Szczecin, Poland; 2 Department of Experimental and Clinical Pharmacology, Pomeranian Medical University in Szczecin, Powstańców Wlkp. 72, 70-111 Szczecin, Poland; 3 Department of General Pathology, Pomeranian Medical University in Szczecin, Powstańców Wlkp. 72, 70-111 Szczecin, Poland; 4 Department of Inorganic and Analytical Chemistry, Poznan University of Medical Sciences, Rokietnicka 3, 60-806 Poznan, Poland; 5 Faculty of Food Science and Nutrition, Poznań University of Life Sciences, Wojska Polskiego 31, 60-624 Poznan, Poland; 6 Department of Physiology, Pomeranian Medical University in Szczecin, Powstańców Wlkp. 72, 70-111 Szczecin, Poland

**Keywords:** thymoquinone, melanoma, metastasis, liposomes, apoptosis, cytotoxicity, mitotoxicity

## Abstract

Thymoquinone has been proved to be effective against neoplasms, including skin cancer. Its high lipophilicity, however, may limit its potential use as a drug. Melanoma remains the deadliest of all skin cancers worldwide, due to its high heterogeneity, depending on the stage of the disease. Our goal was to compare the anti-cancer activity of free thymoquinone and thymoquinone-loaded liposomes on two melanoma cell lines that originated from different stages of this cancer: skin-derived A375 and metastatic WM9. We evaluated the proapoptotic effects of free thymoquinone by flow cytometry and Western blot, and its mitotoxicity by means of JC-1 assay. Additionally, we compared the cytotoxicity of free thymoquinone and thymoquinone in liposomes by WST-1 assay. Our results revealed a higher antiproliferative effect of TQ in WM9 cells, whereas its higher proapoptotic activity was observed in the A375 cell line. Moreover, the thymoquinone-loaded liposome was proved to exert stronger cytotoxic effect on both cell lines studied than free thymoquinone. Differences in the response of melanoma cells derived from different stages of the disease to thymoquinone, as well as their different responses to free and carrier-delivered thymoquinone, are essential for the development of new anti-melanoma therapies. However, further research is required to fully understand them.

## 1. Introduction

Melanoma is a malignant neoplasm that arises from melanocytes, the cells that are responsible for the production of melanin, which absorb UV radiation [1]. Cutaneous melanoma is the most common form of the tumor; however, it can also occur on mucosal surfaces, like the digestive tract, uveal tract, or leptomeninges [2]. In 2020, nearly 325 thousand new cases of cutaneous melanoma were diagnosed, ranking the tumor in 19th place of the most common malignancies. Furthermore, cutaneous melanoma was responsible for more than 55 thousand deaths worldwide [3]. UV radiation is the main environmental factor that contributes to the development of this type of cancer [4].

The achievements that have been made in the systematic treatment of melanoma provide the decrease of the death rates of patients in the USA in recent years. Nevertheless, melanoma is responsible for 0.6% of deaths caused by cancer worldwide, emphasizing the need for investigation of novel therapies [3]. The choice of the optimal therapeutic option depends on the neoplasm localization, staging, and somatic mutations in the tumor. For cutaneous melanoma, surgical resection within healthy margin tissue and optional lymphadenectomy remains the best therapeutic method [5,6]. Metastasis or inoperable melanomas require the usage of systematic treatments, with immunotherapy and kinase inhibitors as the core of therapy [7]. Despite the improvement in the treatment results, the inoperable and metastatic melanomas remain a great challenge for clinicians. Novel approaches are needed to increase the current therapies’ efficacy and safety.

Thymoquinone (TQ) is a member of monoterpenes, a group of chemical compounds containing one isoprenoid unit, and it is the main component of the essential oil of Nigella sativa (black seed), which for years has been known to display various beneficial properties. In the human body, it is metabolized to thymohydroquinone or to semiquinone, and the latter presents anti-cancer activities, increasing ROS in cancer cells [8,9]. Although it has been proved that an elevated level (or inappropriate localization) of reactive oxygen species, acting through both genetic and epigenetic mechanisms [10], is associated with the development of certain cancers, e.g., breast cancer [11], increased ROS production in cancer cells may result in their apoptosis [12], as it has been shown for TQ-treated melanoma [13]. To date, multiple studies reported efficacy of TQ against neoplasms, such as ovarian, breast, colon, prostate, liver, cervical, and skin cancers [14,15].

In recent years, even more studies highlight the possible effects of TQ in melanoma. The anti-cancer activity of TQ has been proved to be caused by various mechanisms, including a direct cytotoxic effect, enhancing apoptosis, DNA damage, and ROS formation. These effects have been attributed to the inhibition of Janus kinase 2 (JAK2) and the activator of transcription 3 (STAT3) signaling pathways [13,16], as well as the reduction of the expression of β-catenin, microphthalmia-associated transcription factor (MITF), and tyrosinase, the molecules that are involved in melanogenesis [17]. The effectiveness of TQ has been tested in melanoma cell cultures [13,17] and in murine models [13,16]. In addition to free thymoquinone, TQ-loaded nanoparticles have also been tested in melanoma cells. Ibrahim et al. evaluated the efficacy of TQ-loaded poly(lactic-co- glycolic acid) nanoparticles (TQ-PLGA NPs) in human melanoma cell lines. The nanoparticles were designed to elevate the concentration of TQ in melanoma cells, as the capillaries in tumor have increased permeability, thereby leading to the retention of TQ. The results showed that this method of administration led to high melanoma cell cytotoxicity, thereby highlighting the efficacy of TQ [18]. So far, there have been no reports of the use of TQ-loaded liposomes in melanoma studies, although liposome- and ethosome-encapsulated thymoquinone tested in other cancer cell models showed high stability and improved bioavailability [19], as well as greater toxicity than free TQ [20].

Despite the advances in research into the effects of TQ on malignant melanoma, there have been few reports showing the effect of this monoterpenoid in human melanoma cells. Additionally, there have been no reports comparing its effects in cells derived from the neoplasms coming from different stages of the disease. Therefore, we evaluated the efficacy of TQ on two human melanoma models: cell culture derived from skin neoplasm and cell culture originated from melanoma nodular metastasis. Additionally, we compared the effectiveness of free TQ with the effectiveness of liposome-encapsulated TQ in the cell lines used. The presented study points out the differences and similarities in response to free TQ and TQ-loaded liposome treatment between the two melanoma cell lines.

## 2. Materials and Methods

### 2.1. Cell Culture

A375 (ECACC, Salisbury, UK) and WM9 (Rockland Immunochemicals, Pottstown, PA, USA) human malignant melanoma cell lines were cultured in a high-glucose Dulbecco’s modified Eagle’s medium (Sigma-Aldrich Merck Group, St. Louis, MO, USA) supplemented with 10% fetal bovine serum (Serana, Pessin, Germany), 2 mM L-glutamine (Serana, Pessin, Germany), 1 mM sodium pyruvate (Sigma, Gillingham, UK), and antibiotics (penicillin and streptomycin; Serana, Pessin, Germany). HEM (human epidermal melanocytes) (Cell Applications, San Diego, CA, USA) were grown in Melanocyte Growth Medium (Cell Applications, San Diego, CA, USA). All the cell lines were incubated at 37 °C, 5% CO_2_, in a humidified atmosphere.

### 2.2. Flow Cytometric Analysis of Apoptosis

A375 and WM9 cells (25 × 10^4^ cells/culture dish) were cultured for 24 h and then treated with free TQ (20 and 40 μM) for 48 h. Alternatively, the cells were pretreated with 10 μM Z-VAD-FMK general caspase inhibitor (R&D Systems, Minneapolis, MN, USA) for 1 h, before TQ treatment. Cells treated with DMSO were used as a negative control and those incubated with 1 μM staurosporine (Sigma-Aldrich, St. Louis, MO, USA), as recommended for melanoma cells [21], as a positive control. The level of apoptosis was assessed on living cells using FITC Annexin V Apoptosis Detection Kit I (BD Pharmingen, Franklin Lakes, NJ, USA) following the manufacturer’s specifications. Briefly, a total of 1 × 10^6^ cells were resuspended in buffer supplied by a manufacturer and mixed with 5 µL of FITC Annexin V and 5µL of propidium iodate (PI). The cells were incubated for 15 min at RT in the dark and were analyzed immediately after staining. Binding of fluorescein-conjugated Annexin V and PI was visualized on the LSR II flow cytometer (BD Biosciences, San Jose, CA, USA). A minimum of 1 × 10^4^ cells were analyzed.

### 2.3. Western Blotting

A375 and WM9 cells (25 × 10^4^ cells/culture dish) were cultured for 24 h and then treated with free TQ (5, 10, 20, 40 μM) for 48 h. Cells treated with DMSO were used as a negative control and those incubated with 1 μM staurosporine were used as a positive control. Next, the cells were harvested and lysed in M-PER™ Mammalian Protein Extraction Reagent (Thermo Scientific, Rockford, IL, USA). Total protein concentration was then determined by BCA method (Thermo Fisher Scientific, Waltham, MA, USA). The equal amount of protein (20 µg) was resuspended in 2 × Laemmli buffer (Bio-Rad, Hercules, CA, USA), heated for 5 min at 95 °C, resolved by SDS-PAGE using 4–20% mini-PROTEAN electrophoresis system (Bio-RAD, Hercules, CA, USA), then transferred to PVD membrane (Bio-Rad, Hercules, CA, USA). Expression of total and cleaved poly-ADP ribose polymerase (PARP) was performed using a primary rabbit PARP antibody (Cell Signaling Technology, Danvers, MA, USA), diluted 1:1000 overnight (4 °C), and secondary mouse/rabbit antibody conjugated to horseradish peroxidase (HRP) (Santa Cruz Biotechnology, Dallas, TX, USA) diluted 1:2000 (1 h, RT). The obtained products were detected using the Western Bright Sirius Chemiluminescent Detection Kit (Advansta, San Jose, CA, USA) and bands were subsequently visualized using a UVP camera (UVP BioImaging system EpiChemi 3, Houston, TX, USA). The readings were acquired from three independent experiments. The intensity of chemiluminescence was measured with ImageJ software 1.53c (National Institute of Mental Health, Maryland, VA, USA). Each densitometric value of PARP was normalized to the value of β-actin (rabbit β-actin antibody, 1:1000 dilution; Santa Cruz Biotechnology, Dallas, TX, USA).

### 2.4. Mitochondrial Membrane Potential (ΔΨ_m_) Assessment

A375 and WM9 cells were seeded in 96-well plates (1.5 × 10^3^ cells/well) and cultured in standard conditions for 24 h. After that, the cells were treated with free TQ at 1.25, 2.5, 5, 10, 20, and 40 µM for 48 h. The cells incubated with DMSO (with the concentration not exceeding 0.025%) were used as a negative control and those treated with 100 μM FCCP (a mitochondrial oxidative phosphorylation uncoupler; Abcam, Cambridge, UK) for 4 h were used as a positive control. Mitochondrial membrane potential (ΔΨ_m_) was assessed by using JC-1 mitochondrial membrane potential assay kit (Cayman Chemical, Ann Arbor, MI, USA) according to manufacturer’s protocol. Briefly, JC-1 staining solution was added to the culture wells and incubated with the cells for 30 min. After that, the cells were washed 2 times with the assay buffer and analyzed by the fluorescent plate reader (Infinite 200 Pro, Tecan, Switzerland), with the fluorescence excitation and emission at 535 nm and 595 nm, respectively, for J-aggregates (present in healthy cells). The fluorescence of J-monomers (present in unhealthy cells) was excited at 485 nm and registered at 535 nm. The ratio of fluorescence intensity of J-aggregates to fluorescence intensity of J-monomers was used as an indicator of ΔΨ_m_. The fluorescence readings were acquired from at least 4 independent experiments (each conducted in quadruplicate).

### 2.5. Preparation of TQ in Liposomes

1-palmitoyl-2-oleoyl-*sn*-glycero-3-phosphocholine (POPC) and 1-palmitoyl-2-oleoyl-*sn*-glycero-3-phospho-(1′-rac-glycerol) sodium salt (POPG) were purchased from Avanti Polar Lipids, Alabaster, AL, USA. Liposomes were prepared by a thin lipid film hydration method, described previously here [22]. Thymoquinone (Cayman Chemical, Ann Arbor, MI, USA) and phospholipids dissolved in chloroform were mixed at 1:2:7 molar ratio of TQ/POPG/POPC and evaporated to dryness under reduced pressure. The obtained thin lipid film was hydrated with PBS (pH 7.4) to force encapsulation, and was completely dispersed by vortexing for ca. 10 min. The resulting liposome suspension was passed 21 times through polycarbonate membranes with a pore diameter of 100 nm, using a syringe extruder (Avanti Polar Lipids, Alabaster, AL, USA) to achieve a uniform size distribution. Subsequently, unencapsulated material was removed by centrifugal filtration using Amicon Ultra centrifugal filters with 50 kDa MWCO cut-off. Briefly, liposomes were added to the Amicon filters and centrifuged at ca. 6000 rcf for 45 min. Afterwards, samples were re-diluted with PBS to the initial volume. Liposome samples were stored at 2–8 °C and protected from light. Liposome size and zeta potential were measured at 37 °C using a Zetasizer Nano ZS (Malvern Instruments, Malvern, UK) by dynamic light scattering (DLS) and laser Doppler velocimetry, respectively [23]. Measurements were carried out in disposable folded capillary cells. Per sample, ten measurements were done with a data acquisition time of 10 s each. Measurements were repeated three times. Before the measurements, liposome samples were diluted ten times in PBS.

### 2.6. Gas Chromatography/Mass Spectrometry

For identification of TQ in liposome, a sample (400 μL) was placed in 10 mL vial with addition of 400 μL of methanol and capped with PTFE/silicon septa caps. Then, the vial with the sample was placed in the ultrasonic bath for 10 min in order to release TQ into the headspace. Extraction of volatiles was performed with CAR/PDMS/DVB 2 cm fiber (Supelco, Bellefonte, PA, USA) at 45 °C over 45 min using CTC combipal autosampler (Agilent Technologies, Santa Clara, CA, USA). The chemical compounds were identified using comprehensive gas chromatography mass spectrometry system—GCxGC-ToF-MS (Pegasus 4, LECO, St. Joseph, MI, USA). The GC was equipped with a SLB-5 column (30 m × 0.25 mm × 0.25 μm) and SPB-50 (1 m × 0.25 mm × 0.25 μm) as a second column. Analyses were run in programmed temperature: initial oven temperature 40 °C was maintained for 1 min, then increased by 6 °C/min to 200 °C and finally 25 °C/min to 235 °C (maintained for 5 min). Second oven temperature was kept 30 °C higher than that of the primary oven and programmed parallel to the first oven. For two-dimensional analysis modulation (liquid N modulator by ZOEX, Houston, TX, USA), time was set at 4 sec and mass spectra were collected at a rate 150 scans/sec. The transfer line was heated up to 280 °C, and the ion source was heated to 220 °C, respectively. For SPME fiber desorption, 260 °C temperature was used with split 20:1 injection. TQ was identified by a comparison of mass spectra with the NIST (The National Institute of Standards and Technology) library (Gaithersburg, MD, USA) and the respective standard. For quantitation purposes, external calibration was prepared by dissolving TQ in 400 mL of methanol and performing SPME extraction, as described above. The calibration curve range was from 100 to 2000 mg/L. Linearity for the standard curve was calculated as the regression coefficient (r^2^) which equaled to 0.987. The calculation was done with Chroma TOF software (LECO, St. Joseph, MI, USA).

### 2.7. Antiproliferative Activity/Cytotoxicity Assay

The antiproliferative/cytotoxicity activity of free TQ and TQ in liposomes was evaluated using the Cell Proliferation Reagent WST-1 assay (Roche Diagnostics, Mannheim, Germany). In the present study, A375 and WM9 cells were seeded in 96-well plates (1.5 × 10^3^ cells/well) and then cultured in standard conditions. After 24 h the culture medium was removed and replaced with fresh medium containing thymoquinone dissolved in DMSO (Sigma-Aldrich, St. Louis, MO, USA) at final concentrations equal: 1.25, 2.5, 5, 10, 20, and 40 µM, or TQ in liposomes suspended in PBS (final concentration: 1.25 μM and 2.5 μM), for 48 h. HEM (1 × 10^4^ cells/well) were incubated for 24 h in standard conditions and then treated with free TQ (final concentrations: 5, 10, 20, and 40 μM) for another 24 h. The cells without tested compounds (but in the medium with DMSO) were used as control, and tested compounds in the medium without cells as blank. The final concentration of DMSO did not exceed 0.025% in any of the plate wells. Afterwards, WST-1 reagent was added and then incubated with the cells for 30 min and absorbance was measured at 450 nm (with 620 nm background correction) using a spectrophotometric microplate reader (Infinite 200 Pro, Tecan, Switzerland). The cell viability was calculated using the following formula: [(A_test_ − A_blank_)/(A_control_ − A_blank_)] × 100%. The readings were acquired from at least 4 independent experiments (each conducted in quadruplicate).

### 2.8. Statistical Analysis

Data are presented as individual samples or mean ± standard deviation. Spearman’s rank correlation coefficient was used to evaluate the associations between TQ concentration and its effects. Differences between the TQ-treated and control groups, as well as differences between the two cell lines studied, were assessed by Mann–Whitney U test. A *p*-value < 0.05 was considered statistically significant. Statistical analysis was performed with the use of Statistica 13.1 (Statsoft, Round Rock, TX, USA).

## 3. Results

### 3.1. TQ Induces Apoptosis in A375 Cell Line More Effectively than in WM9 Cells

**Figure 1:**
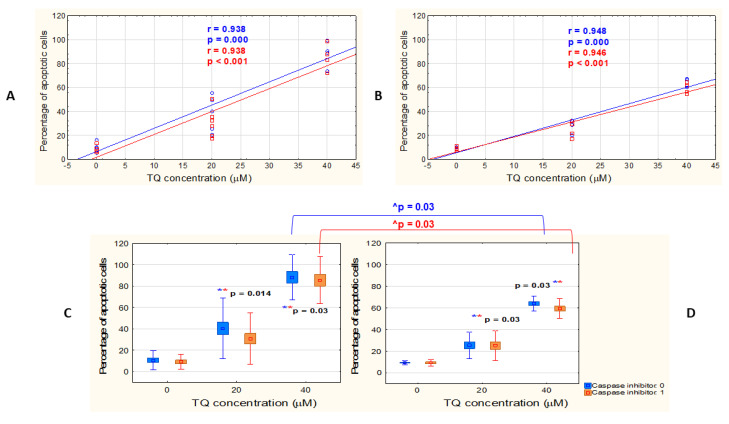
TQ induces apoptosis in A375 and WM9 melanoma cells in a dose-dependent manner. (**A**–**D**): Cytometric analysis of apoptosis in two melanoma cell lines: A375 (**A**,**C**) and WM9 (**B**,**D**) treated for 48 h with different concentrations of free TQ (blue curves and boxes), or with free TQ, following all-caspase inhibitor pretreatment (red curves and boxes). (**A**,**B**): Spearman correlation analysis of the data shows the relationship between TQ concentration and percentage of apoptotic cells. Spearman correlation coefficient (r) and p-value were indicated. (**C**,**D**): Increase in the percentage of apoptotic cells in relation to DMSO control (presented as 0 μM TQ concentration); * p denotes statistically significant differences between the TQ-treated cells and DMSO control (Mann–Whitney U test); ^ denotes statistically significant differences between the two analyzed cell lines (Mann–Whitney U test). Data are shown as mean ± SD; *p* < 0.05 was considered statistically significant.

To assess the response of the different types of melanoma cells to free TQ, A375 and WM9 cell lines were treated with 20 and 40 μM TQ in DMSO for 48 h. The concentrations were chosen based on previously published reports [13], from which we selected two concentrations that caused a noticeable detachment of cells in our microscopy observations (data not presented). The percentage of apoptotic cells was assessed, based on the exposure of phosphatidylserine on the outer plasma membrane, by flow cytometry. Our analysis revealed a very strong positive relationship between the concentration of TQ and the percentage of apoptotic cells in both studied cell lines (Figure 1A,B). Additionally, a significant increase in the amount of apoptotic cells, as compared to the DMSO control (0 μM TQ), was revealed in the both studied cell lines treated with 20 μM TQ and with 40 μM TQ (Figure 1C,D). No significant differences were observed between all-caspase inhibitor-pretreated and inhibitor-untreated cells in the percentage of apoptotic cells, after treatment with 20 μM TQ and 40 μM TQ (Figure 1A,B). However, such differences were noticed between the two cell lines treated with 40 μM TQ, with greater sensitivity to TQ detected for A375 cells, regardless of the presence or absence of the all-caspase inhibitor (Figure 1C).

**Figure 2:**
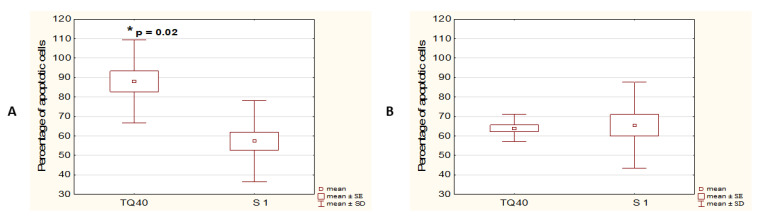
A375 cells showed higher sensitivity to 40 μM TQ compared to staurosporine. Cytometric analysis of apoptosis in A375 (**A**) and WM9 (**B**) cells, treated with 40 μM TQ, or 1 μM staurosporine, for 48 h. * denotes a significant increase in the percentage of apoptotic cells compared to staurosporine control (Mann–Whitney U test); data are presented as mean ± SD; * *p* < 0.05 was considered to be statistically significant.

In order to compare the sensitivity of both studied cell lines to TQ with their sensitivity to a known apoptosis stimulator, the cells were treated with 1 μM staurosporine, an inhibitor of protein kinases, for 48 h. A significantly higher percentage of apoptotic cells was shown for the population of the cells treated with 40 μM TQ than for staurosporine-treated cells, in the A375 cell line, but not in WM9 cells (Figure 2A,B).

**Figure 3:**
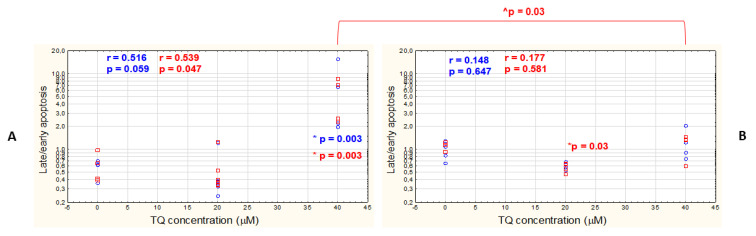
40 μM TQ increases late/early apoptotic ratio in A375 cell line, regardless of the presence or the absence of the all-caspase inhibitor. Cytometric analysis of apoptosis in A375 (**A**) and WM9 (**B**) cells incubated with different concentrations of free TQ (blue circles), or with free TQ following all-caspase inhibitor pretreatment (red rectangles). Spearman correlation analysis of the data shows the relationship between TQ concentration and late/early apoptosis ratio. Spearman correlation coefficient (r) and *p*-value were indicated. Data are presented as mean ± SD; * denotes significant differences in late/early apoptosis between the TQ-treated cells and DMSO control (shown as 0 μM TQ concentration) (Mann–Whitney U test); ^ denotes significant differences between the two analyzed cell lines (Mann–Whitney U test); all the results were considered statistically significant when *p* < 0.05.

As far as the apoptotic stages are concerned, the late/early apoptosis ratio was significantly higher only in the A375 cell line treated with 40 μM TQ when compared with the DMSO control, whether or not the all-caspase inhibitor was used (Figure 3A). Regarding WM9 cells, a statistically significant decrease in the late/early apoptosis was found in the 20 μM TQ-treated cell population pretreated with Z-VAD-FMK (Figure 3B). When comparing the two melanoma cell lines, it was observed that the late/early apoptosis ratio was significantly higher in the inhibitor-pretreated TQ40 μM-treated A375 cells than in WM9 cell line. No significant correlation was observed between the late/early apoptosis and TQ concentration in WM9 cells, regardless of the presence or the absence of the inhibitor, whereas a moderate positive relationship was revealed in inhibitor-pretreated A375 cells (the results were on the borderline of statistical significance).

**Figure 4:**
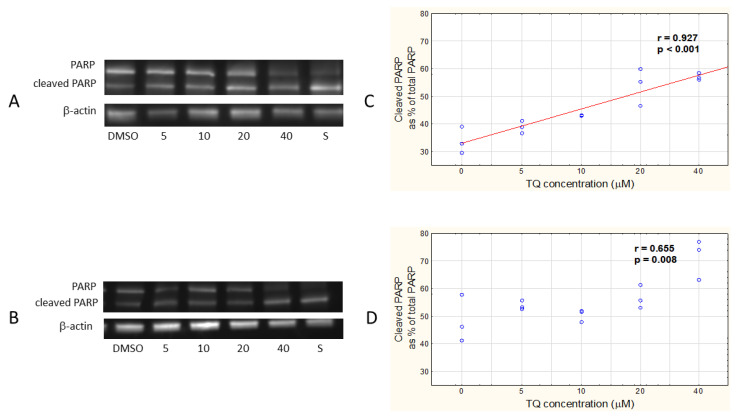
TQ increases the amount of cleaved PARP in A375 and WM9 cells. Expression of PARP detected by Western blot technique in A375 (**A**) and WM9 (**B**) after the treatment with 5, 10, 20, and 40 μM TQ, and 1 μM staurosporine (marked as S) for 48 h. Densitometric analysis of Western blot results showing relationship between the free TQ concentration and the amount of cleaved PARP (Spearman correlation analysis) in A375 (**C**) and WM9 cells (**D**). DMSO control shown as 0 μM TQ concentration; Spearman correlation coefficient (r) and *p*-value were indicated; data are presented as mean ± SD; *p* < 0.05 was considered statistically significant.

To compare the two tested melanoma cell lines in terms of the presence of apoptosis-associated proteins, we examined the expression of the cleaved PARP, an enzyme characteristic of the late stage of apoptosis, in the cells incubated with 5, 10, 20, and 40 μM TQ, by using Western blot (Figure 4A,C) and subsequent densitometric analysis (Figure 4B,D). A very strong positive relationship between the expression of cleaved PARP and TQ concentration was revealed in A375 cells (Figure 4A) and a strong one in the WM9 cell line (Figure 4B). There were no significant differences in the PARP expression observed between the tested cell lines treated with particular concentrations of TQ.

### 3.2. TQ Disrupts Mitochondrial Function in Both A375 and WM9 Cells

**Figure 5:**
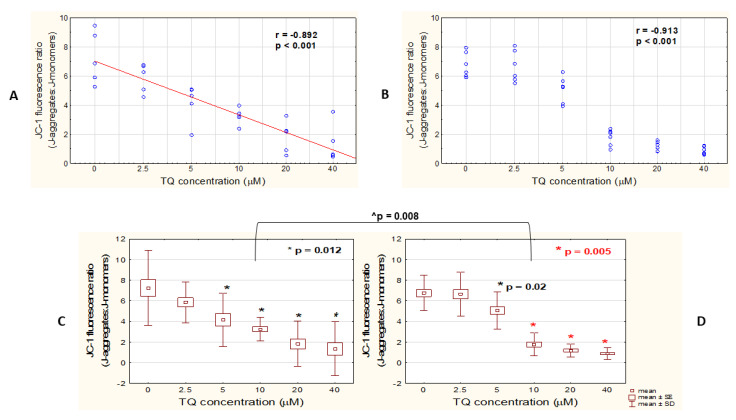
TQ triggers mitochondrial membrane depolarization in A375 and WM9 cells. Spectrofluorometric assessment (JC-1) of the mitochondrial membrane potential (ΔΨ_m_) in A375 (**A**,**C**) and WM9 (**B**,**D**) melanoma cells treated with different concentrations of free TQ for 48 h. The decrease in ΔΨ_m_ is expressed as a drop in J-aggregates/J-monomers ratio. (**A**,**B**): Spearman correlation analysis of the data shows the relationship between the concentration of free TQ and ΔΨ_m_. Spearman correlation coefficient (r) and p-value are shown. (**C**,**D**): Decrease in ΔΨ_m_ in relation to DMSO control (presented as 0 μM TQ concentration). * denotes significant differences between the TQ-treated cells and control (Mann–Whitney U test); ^ denotes significant differences between the two analyzed cell lines (Mann–Whitney U test); data are presented as mean ± SD; all the results were considered statistically significant when *p* < 0.05.

To evaluate whether free TQ similarly affects the mitochondrial function of both studied melanoma cells, A375 and WM9 cell lines were treated with five different concentrations of TQ (2.5, 5, 10, 20, and 40 μM) for 48 h, or with 100 μM FCCP, an oxidative phosphorylation uncoupler, for 4 h (as recommended by the FCCP producer), as a positive control. Mitochondrial function was assessed by using JC-1 dye, a mitochondrial membrane potential (ΔΨ_m_) indicator. In healthy mitochondria, with normal ΔΨ_m_, JC-1 is present as J-aggregates showing red fluorescence; whereas in unhealthy or apoptotic cells with low ΔΨ_m_, the dye remains in monomeric form (J-monomers) that fluoresces in green. Consequently, the drop in the red/green fluorescence ratio indicates mitochondrial depolarization [24]. In our study, a significant very strong negative relationship was revealed between TQ concentration and ΔΨ_m_ in both studied cell lines (Figure 5A,B). What is more, the decrease in ΔΨ_m_ was proved to be statistically significant for both A375 and WM9 cells treated with 5, 10, 20, and 40 μM TQ, when compared to the DMSO control (Figure 5C,D). When comparing the responses of the two melanoma cell lines to individual TQ concentrations, a greater decrease in ΔΨ_m_ was observed in WM9 cells treated with TQ10 than in A375 cells.

**Figure 6:**
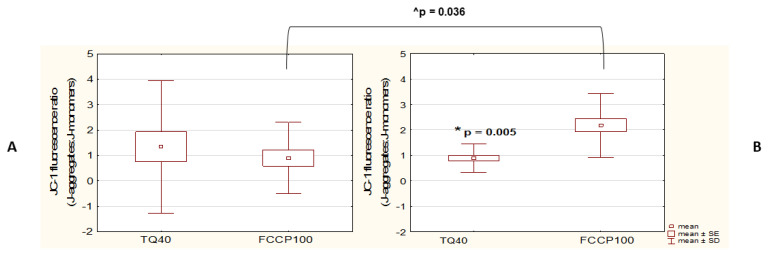
WM9 cell line shows higher sensitivity to 40 μM TQ than to FCCP. Spectrofluorometric assessment (JC-1) of the mitochondrial membrane potential (ΔΨ_m_) in A375 (**A**) and WM9 (**B**) cells treated with 40 μM TQ for 48 h, or with 100 μM FCCP, for 4 h. * denotes a significant decrease in ΔΨ_m_ compared to FCCP control; ^ denotes significant differences between the two analyzed cell lines; data are presented as mean ± SD; *p* < 0.05 was considered to be statistically significant (Mann–Whitney U test).

Additionally, when compared to FCCP positive control, the decrease in ΔΨ_m_ was shown to be significantly higher in the cells treated with 40 μM TQ than in those treated with 100 μM FCCP in the WM9 cells (Figure 6B). Such a relationship was not observed in the case of the A375 cell line, where no statistically significant difference in ΔΨ_m_ between TQ40- and FCCP-treated cells was detected (Figure 6A). However, the observed greater drop in ΔΨ_m_ following the incubation with TQ40 than after FCCP administration in WM9, as compared to A375, proved to be the effect of a significantly lower sensitivity to the uncoupler of WM9 cells.

### 3.3. TQ Concentration Negatively Correlates with the Viability of Both A375 and WM9 Cells

**Figure 7:**
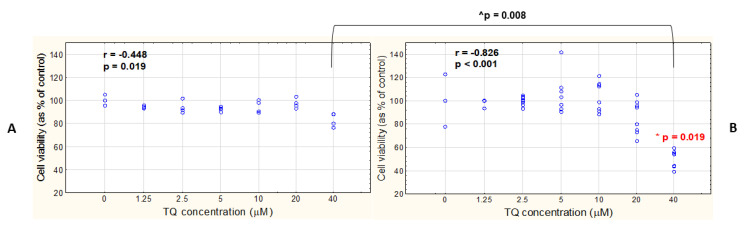
WM9 cells show higher sensitivity to antiproliferative effect of TQ. Assessment of cytotoxicity (WST-1) in the two melanoma cell lines: A375 (**A**) and WM9 (**B**) treated with different concentrations of free TQ for 48 h. Spearman correlation analysis of the data shows the relationship between TQ concentration and cell viability. Spearman correlation coefficient (r) and *p*-value are shown; * denotes significant differences between the TQ-treated cells and DMSO control (presented as o μM TQ concentration) (Mann–Whitney U test); ^ denotes significant differences between the two analyzed cell lines (Mann–Whitney U test); data are presented as mean ± SD; all the results were considered statistically significant when *p* < 0.05.

To evaluate the general viability of free TQ-treated A375 and WM9 lines, the cells were incubated with 1.25, 2.5, 5, 10, 20, and 40 μM TQ for 48 h, and then the cytotoxicity/antiproliferative activity of TQ was assessed with the use of Cell Proliferation Reagent WST-1 assay. The WST-1 test is based on the reduction of the tetrazolium salt to formazan, the amount of which is directly correlated to the number of metabolically active cells. A strong negative relationship between TQ concentration and cell viability was revealed for WM9 cells (Figure 7B), whereas only a moderate negative correlation was shown in the case of the A375 cell line (Figure 7A). However, a statistically significant decrease in the percentage of viable cells, compared to DMSO control, was detected only in the case of the WM9 cells treated with 40 μM TQ, and not in the A375 cells. When the two cell lines were compared, WM9 showed a higher decrease in cell viability after treatment with 40 μM TQ than the A375 cells.

### 3.4. TQ-Loaded LPs Exert a Cytotoxic/Antiproliferative Effect at Low TQ Concentrations in Both A375 and WM9 Cells

**Figure 8:**
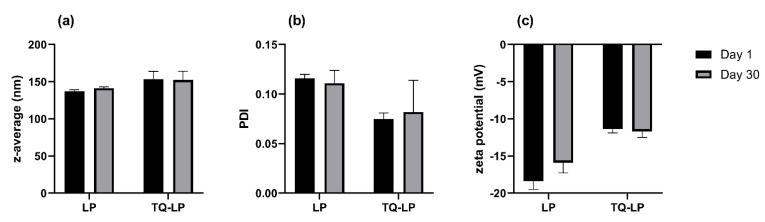
Size (**a**), size distribution (**b**) and zeta potential (**c**) of non-loaded (LP) and thymoquinone-loaded liposomes (TQ-LP) measured on the preparation day (day 1) and following 30 days of storage at 2–8 °C (day 30).

To compare the cytotoxicity of free TQ and TQ-loaded nanocarriers, we prepared TQ in liposomes (TQ-LP) by a thin lipid film hydration method. The parameters describing liposomal formulations obtained for the study are presented in Figure 8 and Table 1. The size of TQ-LP expressed as z-average was 153.4 ± 10.5 nm and was slightly bigger than z_av_ calculated for non-loaded liposomes (141.0 nm). Both samples were characterized by homogeneous size distribution with a PDI of ca. 0.1. The determined zeta potential was −18.4 and −11.4 mV for non-loaded liposomes and TQ-LP, respectively. The negative charge of the liposomal membrane results from the presence of POPG, an anionic phospholipid. The determined values of the zeta potential point out the incipient instability of the system (Kumar and Dixit 2017); however, the measurements repeated 30 days after preparation did not show any particular changes in the liposome size or zeta potential during storage (Figure 8).

**Figure 9:**
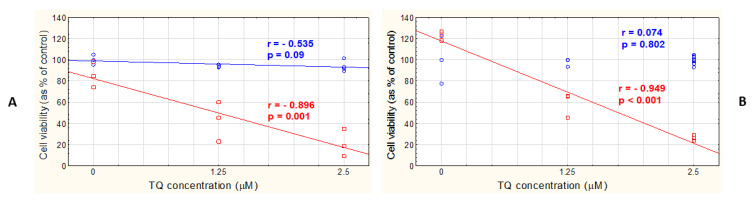
TQ-LP exerts its antiproliferative effect at low TQ concentrations in A375 and WM9 cells. Cytotoxicity assessment (WST-1) in the two melanoma cell lines: A375 (**A**) and WM9 (**B**) treated with low concentrations of free TQ (blue curve and circles), or TQ in liposome (red curves and rectangles), for 48 h. Spearman correlation analysis of the data shows the relationship between free TQ (or TQ in LP) concentration and cell viability. Where 0 denotes DMSO control (blue circles), or an empty liposome control (red squares). Spearman correlation coefficient (r) and *p*-value are shown; data are presented as mean ± SD; *p* < 0.05 was considered to be statistically significant.

The concentration of TQ in the TQ-LP measured by the gas chromatography/mass spectrometry method reached 1100 μg/L. The A375 and WM9 cells were treated with TQ-LP with the final concentration of TQ reaching 1.25 and 2.5 μM after 48 h. For a comparison, both cell lines were also incubated with free TQ (1.25 and 2.5 μM), as well as with a vehicle control (liposome) for the same period of time. A very strong negative relationship between the TQ-LP dose and cell viability was shown in both studied melanoma cell lines (Figure 9A,B: red curves and rectangles). No statistically significant correlation between the dose of TQ and its effect on cell viability was found in the A375 and WM9 cells treated with free TQ (Figure 9A,B: blue curve and circles).

### 3.5. TQ Does Not Affect the Viability of HEM Cells

**Figure 10:**
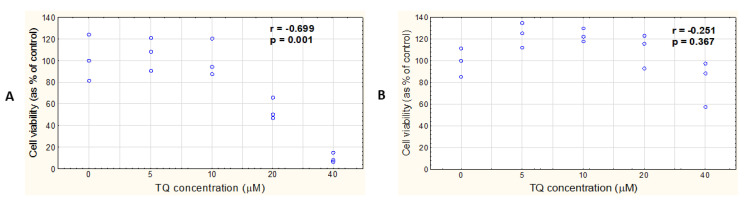
TQ does not inhibit proliferation of normal melanocytes. Cytotoxicity assessment (WST-1) in A375 melanoma cells (**A**) and human epidermal melanocytes (HEM) (**B**) treated with different concentrations of free TQ for 24 h. Spearman correlation analysis of the data shows the relationship between TQ concentration and cell viability. Where 0 denotes DMSO control; Spearman correlation coefficient (r) and p-value are shown; data are presented as mean ± SD; *p* < 0.05 was considered to be statistically significant.

To test whether TQ also exerts its cytotoxic effect on non-cancerous cells, we treated human epidermal melanocytes (HEM) with free TQ (5, 10, 20, and 40 μM) for 24 h. For a comparison, A375 cells were treated analogously. TQ showed a selective antiproliferative effect on A375 cells, with a strong relationship between its dose and cell viability (Figure 10A). No statistically significant correlation between the TQ dose and cell viability was detected in the HEM cell line (Figure 10B).

## 4. Discussion

In the last few years, there has been a renewed interest in compounds of plant origin as potential anti-cancer drugs, or as components of adjuvant therapy [16,25,26,27]. Thymoquinone, long known for its antibacterial, antiviral, and anti-inflammatory properties, has also begun to be analyzed for its anti-cancer properties. Its effectiveness against cancer cells has been well proved on prostate, lung, breast, ovarian, gastric, colon, cervical, liver, and head and neck cancers, as well as against leukemias [15,26,27,28,29,30,31,32,33,34]. There have been several studies on the anti-cancer properties of TQ in the cells of the various types of skin cancer [35,36], a few of which have been devoted to malignant melanoma [37,38]. Most of them, however, have been performed on murine melanoma cell lines, or in mice with a metastasized melanoma [13,16,17].

To complete our knowledge of the effects of thymoquinone on human melanoma cells, we chose two different cell models for our experiments: an A375 cell line, originally isolated from the skin of a patient with malignant melanoma, and WM9 cells, derived from a metastatic site (an axillary node). Our results revealed that free TQ induced apoptosis in a dose-dependent manner, in both analyzed cell lines. Surprisingly, no difference in the percentage of apoptotic cells was observed in the two cell lines treated with TQ following the administration of an all-caspase inhibitor when compared to the cells that had not been pretreated with the Z-VAD-FMK. This, however, might be explained by a possible insufficient caspase blockage by the Z-VAD-FMK, which led to the execution of apoptotic cell death by the low level of active caspases [39]. Although the lack of a response to a caspase inhibitor could also suggest an alternative pathway of TQ-triggered cell death, so called caspase-independent cell death (CICD), the detected apoptosis-related exposure of phosphatidylserine in the outer leaflet of the plasma membrane of TQ-treated cells indicates that such a hypothesis should be considered with caution [40]. What is more, the ineffective blockage of caspases could be also confirmed by the increase in the percentage of early apoptotic cells (decrease in late/early apoptosis) only in the WM9 cell population pretreated with the Z-VAD-FMK, and not in the inhibitor-untreated cells, and the lack of a difference in the response to the highest TQ dose used (40 μM) between the inhibitor-treated and inhibitor-untreated A375 cells. Of the two cell lines tested, A375 was found to be more sensitive to the pro-apoptotic effects of TQ. This may be evidenced by a significantly higher percentage of apoptotic cells in the population of cells treated with TQ40, their higher—in relation to staurosporine—sensitivity to the highest dose of the TQ used and an increased number of late apoptotic cells after incubation with TQ40 in the A375 cell line, when compared to WM9. As shown in previous reports, TQ induced apoptosis in a dose-dependent manner in a B16F10 murine malignant melanoma cell line, with a significant increase in early and late apoptosis after a 24-hour treatment with 60 μM TQ [13], as well as evoked oxidative stress-induced apoptosis in SK-MEL-28, a human skin-derived melanoma cell line [37]. To our knowledge, our study is the first to compare the proapoptotic effects of thymoquinone in human melanoma cells derived from the neoplastic tissues of the various stages of disease.

As reported by Hatiboglu et al., TQ induced apoptosis by the blocking of the Janus kinase 2 (JAK2)/signal transducer and the activator of the transcription 3 (STAT3) pathway and, as a consequence, decreased the expression of anti-apoptotic proteins (Bcl-2 and survivin), and increased the expression of pro-apoptotic proteins, such as BAX and caspase 3, in B16F10 murine melanoma cells, within 24 h [13]. Our studies on human melanoma cells showed that their longer incubation (48 h) with free TQ led to a significant increase in cleaved, and therefore inactive, PARP, in a dose-dependent manner. Cleavage of PARP, a DNA repair enzyme, is a consequence of caspase 3 activation and a hallmark of late apoptosis [41]. Taking this into account, our results showed a similar execution mechanism of TQ-induced cell death as demonstrated previously for mouse melanoma cells. In addition, we showed that, in skin lesion-derived A375 cells, an increase in TQ concentration resulted in an increase in the expression of cleaved PARP, and, thus, the amount of apoptotic cells, while the WM9 cell line (lymph node metastasis) responded much less regularly. This might be explained by the fact that genetically and phenotypically diverse metastatic cells often express differential drug resistance and respond to drug treatment in diversified way [42].

An important part of the middle apoptosis is mitochondrial outer membrane permeability (MOMP) that leads to membrane depolarization [43]. In this study, free TQ triggered mitochondrial membrane depolarization in both tested cell lines, already at the concentration of 5 μM. Additionally, we proved a greater sensitivity of WM9 cells to 10 μM TQ, which resulted in a different shape of the dose–effect curve for these cells, when compared to A375. Although there have been reports showing the mitotoxicity of thymoquinone in cancer cells [44,45,46], only few of them presents its mitotoxic effect in human melanoma studies [37]. Therefore, our study not only confirms the results presented in previous reports, but also highlights the differences in the response of different melanoma cell lines to the mitotoxic effect of TQ.

Regarding general cytotoxicity, TQ was proved to decrease cell viability in a dose-dependent manner in many cancer cell lines, including melanoma [17,47,48]. Jeong et al. showed the reduction of murine melanoma cell (B16F10) viability as a result of 48-hour treatment with TQ (5–30 μM). In our study on human cells, the significant reduction in the percentage of viable cells was registered for 40 μM. In addition, free TQ proved to be more toxic to the metastatic melanoma cell line (WM9) than to the skin-derived one (A375), as evidenced by the higher percentage of viable cells in the A375 line treated with 40 μM TQ than in WM9. This does not seem to be in line with our results, which show a greater sensitivity in the A375 cells to the proapoptotic effects of TQ. These seemingly contradictory findings, however, could suggest the participation of other types of cell death, apart from apoptosis, in the execution of TQ-induced toxicity on WM9 cells [49], which has to be explored in further research.

The great pharmacokinetic advantage of TQ is its ability to penetrate the blood–brain barrier, due to a low molecular weight. However, to reach the adequate bioavailability of TQ, the usage of nanocarriers might be needed, as the molecule has poor water solubility and high lipophilicity [50,51]. Although the use of TQ-loaded nanocarriers has been reported in numerous scientific reports [52,53,54,55], only few of them show the application of such constructs on melanoma cell lines [18]. Ibrahim et al. reported the cytotoxicity of TQ-loaded poly(lactic-co-glycolic acid) nanoparticles (TQ-PLGA NP), with an IC_50_ value between 2.5 and 5 mg/mL, in A375 cells. The TQ-loaded liposomes used in our study proved to have much more cytotoxic properties than free TQ, with the lowest significant cytotoxicity observed at the concentration of 1.25 μM compared to 40 μM for free TQ. Notably, the cytotoxic effect of TQ-LP was shown for both A375 and WM9 cells, with no significant differences between the two cell lines. The results are consistent with previous reports on the use of TQ-loaded nanoparticles proving the lower toxicity of free thymoquinone in relation to the TQ packed in nanocarriers [18]. The lack of noticeable differences in the response to TQ-LP between the studied cell lines might be the result of facilitating the transport of TQ into the cells and requires further research.

Treatment recommendations for melanoma depend on many factors, including surgery, radiotherapy, and chemotherapy (immunotherapy, targeted therapy, etc.), and, usually, assume a systemic drug administration [6]. Recently, topical chemotherapy has been referred to as a promising strategy to improve the conventional treatment of melanoma [56]. With this regard, liposomes can be further considered for either the intravenous, topical, or transdermal delivery of TQ; therefore, slight modifications in liposome composition may be required for further studies, such as PEGylation for iv injections or the addition of penetration enhancers for topical or transdermal applications. To date, there are no reports on the cytotoxic effect of thymoquinone on normal human melanocytes. To complete this knowledge, we attempted a preliminary (one time point) assessment of the toxicity of free TQ in relation to human epidermal melanocytes. We found that TQ showed no significant toxicity to healthy melanocytes within 24 h. To compare the observed effect with the effect of free TQ on melanoma cells, we applied the same TQ treatment length (24 h) to the A375 line. We found a strong positive relationship between the dose of free TQ and its toxicity in A375 cells, which could challenge the previously observed weaker dose–effect correlation with a 48-h TQ treatment in melanoma cells. However, this might be explained by the low stability of TQ in aqueous solutions and a partial loss of its activity during prolonged incubations with cells [50,51]. Nevertheless, further studies comparing TQ activity in melanoma cells and their healthy counterparts at different time points are necessary to confirm our hypothesis.

## 5. Conclusions

In conclusion, TQ proved to be toxic to both melanoma cell lines studied, with no apparent toxicity to normal melanocytes. Although the proapoptotic effect of free TQ was more evident in the A375 cells when compared to WM9, its antiproliferative activity proved to be stronger in the metastatic cell line. The incubation of A375 and WM9 cells with TQ-loaded liposomes resulted in much higher reduction in cell viability compared to free TQ, with no significant differences observed between the responses of the two cell lines. The knowledge of the differences in the response to free TQ and TQ-loaded nanocarriers of melanoma cells derived from different stages of the disease is crucial for the development of effective anti-cancer therapies. Further research using more in vitro melanoma models is needed to explore this topic.

## Figures and Tables

**Table 1:** Studied liposome samples and their composition and characterized parameters: thymoquinone (TQ) load, z-average, polydispersity index (PDI), and zeta potential.

Sample Name	Components	Initial Molar Ratio	TQ Load (µg/L)	Z-Average [nm]	PDI	Zeta Potential [mV]
LP	POPG/POPC	2/8	-	141.0 ± 1.7	0.111	−18.4 ± 1.1
TQ-LP	TQ/POPG/POPC	1/2/7	1100	153.4 ± 10.5	0.075	−11.4 ± 0.5

## Data Availability

The data generated during the current study are available from the corresponding author on request.
